# Supplementary material for: “Falling between the cracks”: Investigating the competing challenges experienced by professionals working with people who hoard
Source: PLoS One. 2025 May 9;20(5):e0323389. doi: 10.1371/journal.pone.0323389 (PMC12063858; doi:10.1371/journal.pone.0323389)
Supplement: S1 File — (PDF) [file pone.0323389.s001.pdf]

Table COREQ

Consolidated criteria for reporting qualitative studies (COREQ): 32-item checklist

No	Item	Guide questions/description
**Domain 1: Research team and reflexivity**		
Personal Characteristics		
1.	Interviewer/facilitator	First and second authors
2.	Credentials	Both have research methods training and experience in qualitative research The first author is a Associate Professor in Psychology and the second author is a doctoral student.
3.	Occupation	Academic researchers
4.	Gender	One female, one male
5.	Experience and training	Both trained in qualitative interview methods
Relationship with participants		
6.	Relationship established	No preexisting relationship with participants prior to the study
7.	Participant knowledge of the interviewer	No prior knowledge
8.	Interviewer characteristics	Both have worked as researchers in mental health and NHS settings
**Domain 2: study design**		

No	Item	Guide questions/description
Theoretical framework		
9.	Methodological orientation and Theory	Inductive, thematic analysis
Participant selection		
10.	Sampling	Purposeful, via a dedicated hoarding forum for professionals
11.	Method of approach	Contact via a hoarding forum that served multi disciplinary professionals in the region. Each interested participant contacted researcher via email
12.	Sample size	35
13.	Non-participation	no one withdrew
Setting		
14.	Setting of data collection	Recruitment via the hoarding forum. Professionals working in a variety of sectors and institutions
15.	Presence of non-participants	No
16.	Description of sample	See description in manuscript (Table 1)
Data collection		
17.	Interview guide	See description on page 5 of manuscript
18.	Repeat interviews	No

No	Item	Guide questions/description
19.	Audio/visual recording	Audiorecording was used to collect the data
20.	Field notes	Yes (reflective diary entries by both interviewers)
21.	Duration	Interviews lasted 25–75 minutes
22.	Data saturation	Yes
23.	Transcripts returned	No
**Domain 3: analysis and findings**		
Data analysis		
24.	Number of data coders	Two primary coders, with full team meeting discussions later in process
25.	Description of the coding tree	Not provided in manuscript
26.	Derivation of themes	Themes were derived from the data
27.	Software	N Vivo
28.	Participant checking	No
Reporting		
29.	Quotations presented	Participant quotations are presented to illustrate the themes / findings, along with an identification for each quotation.
30.	Data and findings consistent	Yes, see results and discussion sections.
31.	Clarity of major themes	Yes

No	Item	Guide questions/description
32.	Clarity of minor themes	Major themes are presented first and secondary themes in a second intention.
