# Supplementary material for: “Falling between the cracks”: Investigating the competing challenges experienced by professionals working with people who hoard
Source: PLoS One. 2025 May 9;20(5):e0323389. doi: 10.1371/journal.pone.0323389 (PMC12063858; doi:10.1371/journal.pone.0323389)
Supplement: Attachment — Submitted filename: Response to Reviewers.docx [file pone.0323389.s004.docx]

# Responses to the editor:

1. Journal additional requirements: Please ensure that your manuscript meets PLOS ONE's style requirements, including those for file naming.

*We have revised the manuscript and referencing to be in line with the PLOS ONE’s style requirements, including for file naming. Please note that we have also updated the citation and reference style to align to the Journal style.*

2. We note that you have indicated that there are restrictions to data sharing for this study. PLOS only allows data to be available upon request if there are legal or ethical restrictions on sharing data publicly. For more information on unacceptable data access restrictions, please see <http://journals.plos.org/plosone/s/data-availability#loc-unacceptable-data-access-restrictions>.

Before we proceed with your manuscript, please address the following prompts:

a) If there are ethical or legal restrictions on sharing a de-identified data set, please explain them in detail (e.g., data contain potentially identifying or sensitive patient information, data are owned by a third-party organization, etc.) and who has imposed them (e.g., a Research Ethics Committee or Institutional Review Board, etc.). Please also provide contact information for a data access committee, ethics committee, or other institutional body to which data requests may be sent.

b) If there are no restrictions, please upload the minimal anonymized data set necessary to replicate your study findings to a stable, public repository and provide us with the relevant URLs, DOIs, or accession numbers. For a list of recommended repositories, please see <https://journals.plos.org/plosone/s/recommended-repositories>. You also have the option of uploading the data as Supporting Information files, but we would recommend depositing data directly to a data repository if possible. We will update your Data Availability statement on your behalf to reflect the information you provide.

In the online submission form, you indicated that [Excerpts of the interview transcripts are included in the findings presented in the paper. However, there are restrictions on access to full interview transcripts, due to issues of confidentiality and anonymity for ethical conduct of qualitative research. The data contains potentially identifying and sensitive personal information and the authors do not have ethical permission to share full interview transcripts as standard. Data requests may be sent to the corresponding author.

All PLOS journals now require all data underlying the findings described in their manuscript to be freely available to other researchers, either 1. In a public repository, 2. Within the manuscript itself, or 3. Uploaded as supplementary information.

This policy applies to all data except where public deposition would breach compliance with the protocol approved by your research ethics board. If your data cannot be made publicly available for ethical or legal reasons (e.g., public availability would compromise patient privacy), please explain your reasons on resubmission and your exemption request will be escalated for approval.

*We note that sharing of full transcripts used in qualitative data remains presently uncommon and controversial largely due to ethical and identification concerns but also on theoretical grounds (including in PLOS ONE), for example as argued Tuval-Mashiach (2021). This is particularly pertinent here as participants are from a well-defined group of specialised professionals in an identified circumscribed geographical location. Nevertheless, we have added openly available meta data on the anonymised data set to FigShare, an online repository,* [*accessible here*](https://aru.figshare.com/account/home?inst=aru#/projects/223014)*. We have included the interview schedule and example anonymised transcripts. Following consultation with the relevant university ethics committee chair, we will make raw anonymised transcripts available to those who contact the research team directly as the risk of identification and harm to participants should be minimised. Our university ethics committee can be contacted via email at* [*se-research@aru.ac.uk*](mailto:se-research@aru.ac.uk)

*Tuval-Mashiach, R. (2021). Is replication relevant for qualitative research? Qualitative Psychology, 8(3), 365–377.* [*https://doi.org/10.1037/qup0000217*](https://psycnet.apa.org/doi/10.1037/qup0000217)

[Anglia Ruskin Research Online (ARRO) - My data (figshare.com)](https://aru.figshare.com/account/home?inst=aru#/projects/223014)

4. Please amend your authorship list in your manuscript file to include author Emma Kaminskiy, Chase Staras, Stuart Brown and Sharon Morein.

*Apologies for this oversight, this has been amended.*

5. Please amend your list of authors on the manuscript to ensure that each author is linked to an affiliation. Authors’ affiliations should reflect the institution where the work was done (if authors moved subsequently, you can also list the new affiliation stating “current affiliation:….” as necessary).

*Affiliations have been included.*

Responses to reviewers

**Reviewer #1:**

This is a timely publication calling the attention of the medical and mainly the helper communities to the problem of hoarding disorder, as well as the unresolved issue of its management. Albeit it is said to be prevalent (around 2% of the general population, more than epilepsy, schizophrenia, autism spectrum disorder and others), there is relatively few literature about it (Pubmed giving 2300 matches, in contrast to several ten-thousands of those above). The literature about its treatment is even more sparse; actually, hardly any (see for instance the excellent review:
Nakao, T. and Kanba, S. (2019), Pathophysiology and treatment of hoarding disorder. Psychiatry Clin. Neurosci., 73: 370-375. [https://doi.org/10.1111/pcn.12853](https://gbr01.safelinks.protection.outlook.com/?url=https%3A%2F%2Fdoi.org%2F10.1111%2Fpcn.12853&data=05%7C02%7Csharon.morein%40aru.ac.uk%7Ce99578b6b4814724f98308dcce80c2db%7C5f35c3da39ae46329ac1afc2f25d2852%7C0%7C0%7C638612299622556959%7CUnknown%7CTWFpbGZsb3d8eyJWIjoiMC4wLjAwMDAiLCJQIjoiV2luMzIiLCJBTiI6Ik1haWwiLCJXVCI6Mn0%3D%7C0%7C%7C%7C&sdata=zZxbUt7QnZvoASj%2Fl%2B7TNWHU44MSOXKxEeylfjDG6aM%3D&reserved=0) ); showing our helplessness in managing this problem and treating those experiencing the disorder; the main difficulty being the lack of insight those affected.

*Thank you for the positive feedback. We agree there is a paucity of research in this area. We have incorporated the additional suggested reference as reference 5 and added this as a citation to paragraph 2, page 3*.

There is a lack by those with hoarding disorder recognizing the pathologic nature of their "collecting passion" (reaching sometimes the degree of lack of capacity), versus a contradictory nature of helpers' intervening into private lives of people with dubious diagnoses, and having in some cases traumatic backgrounds; versus individual and public needs of safety; raising the need of consideration and possibly, legal approach together with harmonizing the multidisciplinary cooperation.
The authors face and show these problems categorizing them into thematic groups, and, in a big work, elaborating 35 unstructured interviews (lasting for an average of 45mins) with those helpers- volunteers or statutory employees. This great effort is impressing, and at the same time, giving a real-word approach of the difficulties tackling the disorder. The authors do not aim to deal with the controversial disorder itself, but through the interviews, one may have some insight into it as well, allowing to see the problem in its complexity. It is written in excellent English (based on my humble knowledge of the language).

*Thank you for the supportive and thoughtful comments.*

I think this work is perfect in its our way, highlighting the difficulties in narrative texts. This is a somewhat structured way of discussing the issue and calling attention to it. This is refreshing in a way- no statistical calculations, probabilities and significances; just real word experiences and comments of those involved. At the same time we cannot call it a 'scientific' paper, and the conclusion can be the same as the induction: hoarding is a complex, multifaced condition, hard to tackle and needing multidisciplinary approach.
Considering this point, the paper should not be published; on the other hand, it should be published because of the paucity of hoarding literature, because it calls attention to this difficult multiangle problem may be evoking further comments and suggestions including psychiatric treatment and authorities' careful intervention of management.
For the latter reason and hope, I suggest to publish the paper.

*Thank you. Qualitative methodology was chosen for this study due to the lack to established theory and complexity of current practices in the field. Consequently, while we did not aim to test explicit hypotheses, we did follow established scientific protocol for qualitative research. To strengthen the reporting of this work, we have now included the completed checklist reporting the consolidated criteria for reporting qualitative studies (COREQ) as supplementary material.*

**Reviewer #2:**

The authors provide a sound argument. The study also includes a diverse group of professionals to appreciate the challenges they face from their perspective. Given that the participants were all recruited from the attendees of a regular forum to discuss hoarding, it is interesting to know that there were varied levels of understanding of hoarding and ways to manage the situation. If possible, the researchers can follow up with a debriefing or presentation at these forums to further emphasize the need for multi-disciplinary action and a consensus on the lengths and breaths of hoarding.

*Thank you for the thoughtful comments and the positive feedback. We thank the reviewer for suggesting further follow up work with the forum. Indeed, we plan to undertake further work with members of the forum and other practitioners that will be in line with this suggestion.*

**Reviewer #3:**

Thank you to the authors for the opportunity to review this paper. The need for this work is evident and provides greater awareness for understanding how we can support this under-represented population. The paper is overall well written and provides a compelling picture of the difficulties in working with hoarding populations. I believe it will be very useful in improving our care for hoarding within the community. There are some minor changes and recommendations made below. I hope they prove to be useful.

*Thank you for the supportive feedback and the positive comments. We have incorporated your suggestions and believe this has improved the paper – thank you.*

- In the introduction, the first sentence should be supported with a reference, perhaps Timpano et al. (2013)
Timpano, K. R., Broman-Fulks, J. J., Glaesmer, H., Exner, C., Rief, W., Olatunji, B. O., ... & Schmidt, N. B. (2013). A taxometric exploration of the latent structure of hoarding. Psychological assessment, 25(1), 194.
- Second sentence should have a comma after “when this becomes extreme”

*We have corrected the typographical issues and including additional citations to Timpano et al. (2016) as reference 1.*

*Timpano KR, Muroff J, Steketee G. A Review of the Diagnosis and Management of Hoarding Disorder. Curr Treat Options Psych. 2016 Dec 1;3(4):394–410.*

- In the introduction, it states that early intervention is less likely, possibly related to reduced insight and the stigmatising nature of the condition. Beyond this, it could be mentioned that the criteria for hoarding (in particular the clutter criterion) usually requires decades of accumulation of items before it is met. As such, many individuals distressed by object attachment would not meet criterion until hoarding behaviours become extreme, necessitating multiagency support.

*We have added additional wording in paragraph 2, page 3 and make reference to the clutter criterion impacting likelihood early intervention.*

- There could be a greater rationale for the necessity of MDTs in working with hoarding populations. For example, different professionals are involved due to the common fire risks, hygiene concerns, risk of losing housing, psychological treatment, safety mitigation, etc.

*We amended paragraph 3, page 3, to provide more rationale for MDT approaches for hoarding.*

- Typo on page 4: diverse backgroundS

*This has been amended*

-The results were very interesting in that many of the opinions of the professionals are well-aligned with emerging literature in hoarding populations. For example, professionals noted that hoarding groups had specific complexity including complex social needs, a history of trauma, and use possessions to replace social interaction. These are directly aligned with emerging social impairment models of hoarding (e.g., Chen et al., 2022), which could be noted within the discussion.

This may suggest that professionals struggle with these issues because they are consistently observed social difficulties within this population, and therefore these results support the research within the hoarding space, which has suggested creating interventions that target these social difficulties too (e.g., David et al., 2022).

Chen, W., McDonald, S., Wearne, T., & Grisham, J. R. (2022). Interpersonal functioning in hoarding: An investigation of the link between hoarding symptoms and social support, social anhedonia, and social rewards. Journal of Affective Disorders Reports, 8, 100313.
David, J., Crone, C., & Norberg, M. M. (2022). A critical review of cognitive behavioural therapy for hoarding disorder: How can we improve outcomes?. Clinical psychology & psychotherapy, 29(2), 469-488.

*Thank you for the additional references and for raising these points. We have made revisions to incorporate these in paragraph 2, page 16, and paragraph 2, page 17 with Chen et al., (2022) as reference 35 and David et al., (2022) as reference 38.*

- The discussion was interesting and synthesised the results well, including providing interesting recommendations for the field. Suggestions are as follows:
- On page 16, it could be useful to include references of papers outlining biopsychosocial understandings of hoarding on page 16, e.g., Mathes et al. (2022)
Mathes, B. M., Timpano, K. R., Raines, A. M., & Schmidt, N. B. (2020). Attachment theory and hoarding disorder: A review and theoretical integration. Behaviour research and therapy, 125, 103549.

*Thank you for these suggestions. We have provided additional citation to Mathes (2020) work (Page 16, paragraph 2) as reference 34.*

- Regarding evidence-based interventions, Buried in Treasures is a standard intervention that could be recommended within the discussion. There is evidence to support it’s utility as a gold-standard CBT based protocol for hoarding, and has been found to be effective when conducted by mental health professionals, in support groups, or as a self-help program. Perhaps this could be mentioned in pages 16-17 when discussing how to involve and target psychological factors in hoarding.

*Thank you – we have incorporated reference to Buried in treasures (Page 17, paragraph 2) as references 41 and 42 and to harm reduction approaches (Page 17, paragraph 3) as reference 45.*


- On page 17, the authors discuss the need to provide clear or dedicated care pathways to support individuals who hoard. As a broad stroke recommendation, findings (including from the literature on harm reduction approaches; Tompkins, 2011) may suggest that targeting safety and harm minimisation is usually a top priority, followed by prioritising psychological intervention. Notably, once bare minimum harm minimisation is met, harm reduction approaches take up to 2 years, suggesting that multiple concurrent intervention may be useful too, including further harm reduction with psychological intervention.

Tompkins, M. A. (2011). Working with families of people who hoard: a harm reduction approach. Journal of clinical psychology, 67(5), 497-506.

*Indeed the reviewer raises an excellent point as we had neglected to explicitly refer to harm reduction approaches in the study. The discussion has been amended (page 17) to address this including this citation as reference 45.*

- Typo page 17: “dedicated care pathways TO support”

*This has been amended*

- This may not be a question that can be answered, but one that comes up in the context of this research, so I’m curious about the author’s thoughts. Regarding recommendations, it is useful to suggest care pathways that incorporate multi-agency collaboration. However, there are limitations to doing this, including agencies being under-resourced in being able to train professionals on hoarding specifically, and hoarding not being recognised as a top priority by government policies, and thus not attracting significant grant funding to improve research as well. Would the authors have additionally suggestions about how this could be targeted over time?

*The present study indeed points specifically to some of the limitations and challenges involved in implementing multi-agency collaborations for hoarding in practice. We believe the greater challenges the reviewer raises are outside the scope of the present study but we have been working on addressing precisely these issues of how to improve training, support provision and understanding. To date, in consultation with others across the UK, one option has been to make an economic or financial argument (e.g., how much spending could be saved by local authorities, including human cost). This has met with local success in some places. Given the lack of commissioning for hoarding by the NHS or the absence of dedicated NICE guidelines, we have considered approaches that complement traditional care pathways such as via families. Another approach has been in greater coordination and collaboration across the UK, which has led to the establishment of the UK hoarding research network aimed at researchers and the UK hoarding partnership aimed at professionals and those personally affected by hoarding, with the two complementing and informing each other.*
