# Supplementary material for: “Falling between the cracks”: Investigating the competing challenges experienced by professionals working with people who hoard
Source: PLoS One. 2025 May 9;20(5):e0323389. doi: 10.1371/journal.pone.0323389 (PMC12063858; doi:10.1371/journal.pone.0323389)
Supplement: Attachment — Submitted filename: Re-review hoarding.docx [file pone.0323389.s003.docx]

Re-review of the paper

**’Falling between the cracks. Investigating the competing challenges experienced by professionals working with people who hoard.”**

This remains a stopgap paper, dealing with the extremely severe and disregarded issue of hoarding disorder.

The authors review the difficulties of working with people who hoard and do not attend to the important question - what kind of disorder is it, and consequently, how could we treat it. At the same time, indeed, they stand to their word given in the title- dealing just with the challenges of managing the problem; however, it would be nice to know some more about the condition itself- maybe a too ambitious expectation at this point.

One have to esteem the authors’ efforts in addressing this burning but neglected field. They have followed reviewers’ suggestions, just a few typos remained.

For the latter reason I suggest ’minor revision’; but I propose to publish the paper in the hope of evoking further ones on the nature and treatment of hoarding.
